# Extended difficulties after psychedelic experiences: Prevalence and associations in a global, multilingual sample

**DOI:** 10.21203/rs.3.rs-9304959/v1

**Published:** 2026-04-08

**Authors:** Oliver, C. Robinson, David Luke, Jules Evans, Jacob, S. Aday, Matthew Johnson, Stephanie Lake, Daniel Kruger, Kevin, F. Boehnke, Philippe Lucas

**Affiliations:** Regen’s University London; University of Greenwich; Challenging Psychedelic Experiences Project; University of Michigan–Ann Arbor; Johns Hopkins University; University of California, Los Angeles; University at Buffalo, State University of New York; University of Michigan–Ann Arbor; University of Michigan–Ann Arbor

**Keywords:** psychedelics, adverse reaction, extended difficulties, challenging experiences, Global Psychedelic Survey

## Abstract

As psychedelic use increases and integrates with mainstream medicine, understanding the prevalence and nature of postpsychedelic adverse outcomes is critical. We investigated extended difficulties after psychedelic use via the Global Psychedelic Survey (GPS) 2025, which is a multilingual online survey of people who have used psychedelics. Data on the prevalence, characteristics, and associations of retrospectively reported extended difficulties was gained from N = 6,476 participants. The most common difficulties reported were existential struggle (36.6%), depression (34%), and derealization (29.4%). Existential struggle was rated as the most severe difficulty, but also the one contributing most to healing. Overall, 48.3% of participants reported one or more difficulties lasting 24 hours or more, and 9.9% experienced difficulties for over a year. Clinically relevant disruptive difficulties (defined as leasting at least a month that caused disruption to daily life) were reported by 8% of the sample and were associated with younger age at the time of survey and at first psychedelic use, lower income, lack of family support, lower emotional stability/conscientiousness/agreeableness, higher pre-existing anxiety/depression, and using psychedelics to treat mental health conditions. The results underscore the necessity for evidence-based education on the potential risks and benefits of psychedelics, robust safety guidelines in clinical psychedelic interventions, and additional services for those experiencing post-psychedelic extended difficulties.

## Introduction

As the therapeutic use of psychedelics continues to move closer to mainstream medicine, researchers are increasingly investigating adverse reactions and extended difficulties that persist after the psychedelic experience ^1,2,3, 4^. These studies are revealing a complex landscape of long-term psychological and perceptual challenges in a minority of people who use psychedelics.

Few studies have looked at the prevalence of extended difficulties in psychedelic users, and those that have been done have been single-country samples. In terms of prevalence rates across single countries, a representative survey of 2822 U.S. adults found that 9% of classic psychedelic users experienced functional impairment lasting longer than one day, while 3% sought professional medical or psychiatric help for these doffoculties.^5^ Also, Olofsson et al.^4^ found that 6% of a large survey of US adult psychedelic users reported post-acute difficulties lasting longer than one day, and 1% experienced difficulties for more than one year. The most frequently reported post-acute difficulties in this study were general anxiety (34%), negative changes in self-concept (26%), and social disconnection (23%).

A survey of 770 Norwegian psychedelic users found that 23% reported persistent adverse events, with 11% reporting the difficulties lasted longer than a week, 25% reporting reactivation of effects and 50% reporting a craving for more psychedelic experiences^6^. Similarly, a survey by Weiss et al. of 218 naturalistic psychedelic use found that 14% of people reported feeling more anxious or depressed for an extended period due to their psychedelic usage, and a third of these people felt these personality changes were permanent and unwanted^7^.

In a survey of 426 US military veterans who use psychedelics, Davis et al. found that 59% reported adverse events associated with psychedelic use, the most common of which were reactivation of psychedelic effects (33%), craving (26%), and negative impact on relationships (14%). Approximately 18% of these participants reported that they sought therapy because of adverse events related to psychedelic use^8^.

In terms of types and variants of difficulties experienced, recent studies have aimed to develop a taxonomic understanding of these, as well as knowledge of duration and severity of these different difficulty types. In a mixed-methods survey study of 608 individuals who reported extended difficulties after real-world psychedelic usage, finnding that the most prevalent difficulties reported were emotional difficulties (76%), specifically anxiety and fear, alongside existential struggle (50%) and social disconnection (52%), with post-psychedelic difficulties lasting over a year in 30% of participants. Despite these difficulties, nearly 90% of respondents maintained that the potential benefits of psychedelics outweigh the risks in supportive environment^9^. Robinson et al.^10^ investigated duration and severity of prolonged difficulties in a sample of 159 individuals who reported adverse reactions following psychedelic experiences. The most prevalent challenges identified were social disconnection (72%), anxiety and panic attacks (68%), and existential struggle (65%). Although anxiety was rated as the most severe difficulty, existential struggle and diminished self-esteem were the longest-lasting, with durations averaging over 15 months.

Other studies on post-psychedelic difficulties have looked at risk profiles of specific drugs. With ayahuasca, research indicates that while many find the experience positively transformative, approximately 56% of a sample of 10,836 users report adverse mental health effects in the weeks or months following consumption and 12% of users sought professional support for persistent adverse psychological effects. However, 88% of those who did experience adverse effects considered such mental health effects as part of a positive process of growth or integration^11^. A study of 1993 psilocybin users found that 10% reported negative symptoms after psilocybin use that lasted at least one week, and 8% sought professional treatment ^12^. A survey (N = 2833) of naturalistic use of psilocybin by Nayak et al.^13^ found that 11% reported negative persistent effects at 2–4 weeks after use and 7% reported persisting negative effects two months after use. An analysis of data from 20,000 + participants in the Global Drug Survey 2020 found that 22.5% of LSD and/or psilocybin users reported at least one negative post-acute outcome, with 6% reporting difficulties lasting over a month, where LSD was associated with a higher number of negative outcomes compared to psilocybin mushrooms ^14^.

Predictors of post-psychedelic difficulties and adverse reactions include both dispositional and contextual factors. Barrett et al.^15^ found that the personality trait of neuroticism is positively associated with the intensity of challenging experiences. However, Studerus et al.^16^ did not find an association between neuroticism and psilocybin response in laboratory studies (possibly due to exclusion criteria) but did find that high emotional excitability was associated with acute unpleasant or anxious reactions. Conversely, traits like openness and the ability to “surrender” to the experience are protective against acute adverse effects ^17^. Age has been negatively related to the intensity of acute and long-term effects of psychedelics ^18,19^ and negatively associated with unpleasant/anxious reactions to psilocybin (Studerus et al., 2012), with some speculating that heightened 5-HT2A binding potential in younger compared to older adults may underlie blunted responses later in the lifespan ^20^. In terms of contextual factors, Evans et al.^5^ and Robinson et al.^6^ both found that a lack of guidance and containment, such as taking the drug without a guide or in an unstable environment, increases the risk of long-term difficulties. Similarly, Simonsson et al.^7^ identified “no preparation” and “negative mindset” prior to use as key correlates of severe distress. Evans et al.^5^ and Olofsson et al.^4^ both found adverse childhood experiences made challenging psychedelic experiences and extended difficulties more likely. Griffiths et al.^21^ found that high doses in a clinical study are a primary predictor of acute fear and anxiety, which can subsequently lead to extended distress. Bremler et al.^22^ found that unsafe or complex environments during the experience, unpleasant acute experiences, prior psychological vulnerabilities, high- or unknown drug quantities and young age predicted extended post-psychedelic difficulties.

### Rationale, Aims, and Research Questions

The principal aim of this study was to investigate the prevalence, characteristics, and associations of retrospectively reported extended difficulties from psychedelic experiences by way of an analysis of data from the GPS 2025^23^. The central rationale for pursuing this aim is that there is currently little systematic research into extended difficulties following psychedelic use, in studies based on a large international sample. Our primary research questions included:

1. What are the prevalence rates for (a) any kind of extended difficulty, and (b) specific kinds of difficulty experienced after a psychedelic experience, for varying durations spanning from 24 hours to a year or more, for the whole sample and for specific continents, within a global, multi-lingual sample of people who use psychedelics?
2. What associations are shown between extended difficulties of clinical relevance (defined as difficulties enduring a month or more that interfere negatively with daily activities) and demographic / personality / psychedelic usage / social support variables?

## Methods

### The Global Psychedelic Survey

The Global Psychedelic Survey (GPS) 2025 is the latest iteration of an international, multilingual online survey, led by an interdisciplinary team at the Michigan Psychedelic Center at the University of Michigan, working with academic collaborators from over 20 countries. Additionally, 40 NGOs that engage with the psychedelic community were invited to provide feedback on the survey design. The GPS 2025 was delivered in English, Portuguese (Brazilian and Traditional), Spanish, Italian, Japanese, German, Hebrew, French, Ukrainian, Chinese (traditional and simplified), Finnish, Persian, Russian, Dutch, Greek, Arabic, Polish and Turkish. The study was reviewed by the Health Sciences and Behavioral Sciences Institutional Review Board at the University of Michigan (ID: HUM00268282) and was deemed exempt from ongoing IRB overview per exemption 2(i) and 2(ii) at 45 CFR 46.104(d). The full methodology for the survey is described in Lake et al.^23^.

### Recruitment and Sample

Recruitment was initiated through a range of means, including newsletter articles, social media and traditional press interviews, and collaborators posting about the upcoming study via their own channels and email lists, including lists associated with the 40 collaborating NGOs. The survey was also advertised via paid ads on Meta in some jurisdictions. The survey ran from May 1st to May 23^rd^, 2025. Eligibility criteria were (a) being aged 21 years or over, (b) having fluency in one of the 19 available survey languages, and (c) having used a psychedelic substance on at least one lifetime occasion.

The total achieved GPS sample was N=9087. Of these, 6476 (71.3%) completed the questions on extended difficulties, and the remainder dropped out of the survey before completing them. Of these 6476 participants, 3220 were assigned female at birth, and 3247 were assigned male at birth. Participants were born in 120 different countries; the frequencies of participant numbers by country are available in Supplementary Materials. In terms of educational level, 1.8% had less than high school, 16% had high school or equivalent, 41.6% had a university or technical degree, 29.3% had a graduate degree, and 11.3% a doctoral or professional degree. In terms of employment status, 53% were employed full-time, 19.2% employed part-time, 8.1% were students, 7.1% were retired, 2.6% were disabled or unable to work, and 9.8% were unemployed.

### Procedure

Data collection was conducted on Qualtrics, an online survey platform that is HIPAA and GDPR compliant. For the non-English languages, a multi-step strategy of translation and back-translation was used. The survey was anonymous and confidential. Informed consent was gathered online (see Appendix 1 for ICF) and participants who completed the survey could enter a drawing for one of 15 Amazon gift cards (each 100 USD). responses. All resulting email addresses were immediately destroyed following the draw.

### Measures

The full set of questions contained in the GPS is available in Supplementary Materials. Skip logic was employed, meaning that progress through the survey was variable for each respondent. The variables and scales that are reported in this paper are shown in Table 1.

The initial instruction and question on the extended difficulties sub-survey was as follows:

“We’d now like to ask you about potential harms or difficulties that lasted more than 24 hours after the psychedelic drug effect has subsided, which you believe were related to taking a psychedelic substance. The following questions refer to your overall psychedelic experiences, rather than just one specific intense experience. Have you ever experienced the following more than 24 hours after the psychedelic drug effect has subsided?”

This question was followed by a matrix showing a list of difficulties, which participants could endorse on scale as follows:

1) No, 2) Yes – Less than one week, 3) Yes – One week to one month, 4) Yes – More than a month but less than a year, 5) Yes – More than a year. The list of difficulties was as follows:

1. A sense of disconnection from other people
2. Anxiety and/or panic attacks
3. Feelings of depression
4. Existential struggle
5. Derealization
6. Depersonalization
7. Lowered self-esteem
8. Sleep problems inc. nightmares
9. Cognitive confusion or memory issues
10. Visual distortions
11. Disappointment with events after the experience

For each of these difficulties, after the presentation of this initial list, if a person stated that they experienced a difficulty (i.e., endorsed options 2-5), for that difficulty they were shown three follow-up questions. See below, with ‘sense of disconnection from other people’ as the example:

- How severe was your sense of disconnection from other people? (Not at all severe / Somewhat severe / Moderately severe / Very severe / Extremely severe)
- Did your sense of disconnection from other people interfere with your daily life such as (Not at all / Somewhat / Moderately / Very Much / Completely)
- work or personal relationships?
- To what extent do you feel your sense of disconnection from other people was part of an extended healing process? (Not at all / Somewhat / Moderately / Very Much / Completely)

There were two additional questions to conclude the sub-survey:

Is there a specific psychedelic TYPICALLY associated with ongoing difficulties in your personal experience?

- No, none specifically
- Ayahuasca
- DMT (N,N-Dimethyltryptamine)
- 5-MeO-DMT (5-Methoxy-N,N-Dimethyltryptamine)
- Iboga / Ibogaine
- Ketamine (K)
- LSD / Acid
- MDMA / MDA (Ecstasy / Molly)
- Mescaline (San Pedro, Peyote, etc.)
- Nitrous Oxide (non-dental/surgical, e.g., whippets)
- Psilocybin (mushrooms or synthetic)
- Salvia divinorum
- Synthetic phenethylamines (2C-B, 2C-I, DOM, DOI, 25B-NBOMe, etc.)
- Synthetic tryptamines (AL-LAD, ETH-LAD, 4-HO-MET, 5-MeO-MiPT, etc.)
- Other psychedelic drug(s):

Is there a specific setting TYPICALLY associated with ongoing difficulties in your personal direct experience?

- Indoors in a familiar setting (e.g., my home or a friend’s house)
- In a licensed clinic, wellness center or hospital
- At an underground therapist / practitioner’s office or clinic
- At a small gathering / retreat
- At a large public gathering / party / rave / event
- Outdoors in a natural environment (e.g., in a park or forest)
- None of the above

### Data preparation and analysis

For the purposes of investigating associations with reports of extended difficulties that last a month or more and that interfered with daily life, we created a categorical variable scored as 0 or 1, where 1 indicated that the person had (a) experienced any difficulty for more than 1 month, and (b) a mean score of 2 or more for difficulties interfering with daily life, as averaged across all 11 difficulties (2 on the scale equates to ‘Somewhat’ on a 5-point scale of *Not at All / Somewhat / Moderately / Very Much / Completely*). We report Cohen’s *d* effect sizes and Cramer’s *V* effect sizes. We ran a series of *t*- tests and Chi Square tests to explore associations between this variable with the variables specified in Table 1. We focus on effect sizes in our analysis, although we also report *p* values as descriptive information to complement the effect sizes, without a defined alpha cut-off. All analyses reported in this article were conducted in SPSS Version 29.

## Results

Prevalence rates of extended difficulties showed decreases with duration. In terms of *any* kind of difficulty, 48.3% of the subsample reported a difficulty of any duration of 24 hours or more, 40.8% reported any difficulty lasting 24 hours to a week, 31.6% reported any difficulty lasting over a week but less than a month, 18.5% reported any difficulty lasting over a month but less than a year, and 9.9% reported a difficulty lasting over a year. Table 2 contains the percentage of participants who reported each pre-specified difficulty, the percentage who reported any difficulty, plus the percentages who endorsed the four levels of duration. It also shows means for the reported severity, disruptiveness to life, and perceived contribution to an extended healing process of all reported difficulties. The three most reported difficulties were existential struggle (36.6%), feelings of depression (34%), and derealization (29.4%). Existential struggle was also the most common difficulty across three of the four duration points, but feelings of depression were the most common difficulty for the duration of 24 hours to a week. Existential struggle was also rated as the most severe difficulty *and* the difficulty that was perceived to contribute most to healing. The difficulty showing highest level of interference with daily tasks was lowered self-esteem.

Analysis of prevalence difficulties across geographical regions defined by continent shows that all symptom types were reported in each of the five major inhabited continents (see Table 3). Differences in the distribution of these symptoms varied minimally (with multiple very weak to weak effect sizes ranging from Cramer’s *V* = 0.03 to 0.11 for 4df), with the strongest variations across continents being for the difficulty ‘existential struggle’, due to high levels of reported existential struggle in Asia (53.9%) and low levels of depressed feelings in Oceania (25.8%).

Our analysis found that 8% of the sample who completed the extended difficulties sub-survey (N = 547 of 6746) reported difficulties for a month or more that interfered with their daily life. Associations of this variable with other continuous and categorical variables, along with effect sizes, *p* values and degrees of freedom, are reported in Tables 4 and 5. Notably, 42.4% of participants with disruptive difficulties lasting over a month have sought external support to help deal with it, and 79.1% of this group found that support to be effective (see Table 5). In contrast, a lower proportion of other participants (27%) have sought external support, while a much higher percentage (93.6%) of this group found it to be effective.

**Figure 1:**
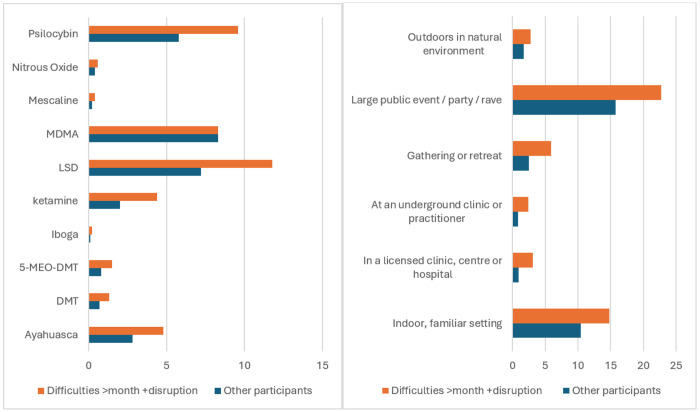
Psychedelics and settings associated with post-experience difficulties in (a) the group reporting disruptive difficulties of a month or more (N=547), and (b) other participants also reporting difficulties (N=5929) Note: % = Percentage of participants who responded to these questions about typical psychedelics and settings. These questions were only shown to participants who reported extended difficulties. Note: Figure 1 does not include negative responses, i.e. those who did perceive a link between psychedelic or setting and their difficulties. These frequencies are follows: No perceived link between difficulties and specific psychedelics = 50.9% of month+ disruptive difficulties group, and 69.7% of other participants. No perceived link with specific settings = 48.3% of month+ disruptive difficulties group, and 68% of other participants.

Figure 1 shows descriptive statistics for the psychedelics and settings perceived to be associated with extended difficulties, stratified by two groups: (1) those who reported disruptive difficulties lasting a month or more, and (2) other participants. This descriptive information expands on the Chi Square and effect sizes shown in the bottom two rows of Table 4.

The significant associations evidenced in Tables 4 and 5 show that those who report *disruptive extended difficulties of a month or more* have the following features: They are more likely to be younger at point of survey completion, and younger when first initiating psychedelic use. They have a lower average income, are more likely to be single, and their family is typically more disapproving of psychedelics. In terms of personality and mental health, they have on average lower Conscientiousness, lower Agreeableness, lower Emotional Stability, and are higher in anxiety and depression symptoms. They are more likely to be using psychedelics to treat a mental health condition and/or a problematic behaviour. In terms of difficulties experienced, those with disruptive extended difficulties showed a higher mean severity and disruption, but also a higher mean perception of contribution of difficulties to healing. These individuals are less supportive of legal access to psychedelics. Overall, Table 5 and Fig. 1 show that they perceive more links between extended difficulties and specific drugs *and* links between extended difficulties and settings, for all drugs (except MDMA) and for all settings. Finally, a much higher proportion of whose with difficulties of clinical relevance sought external support compared with other participants, but they perceived lower effectiveness of this support.

## Discussion

These findings from the GPS 2025 provide important insights for the emerging research fields of post-psychedelic difficulties. A key finding is the high prevalence rates of extended difficulties in people who use psychedelics. Nearly half (48.3%) of respondents experienced some form of difficulty lasting over 24 hours, with a decline as duration increases, and yet 9.9% reporting difficulties lasting over a year. Additionally, the study identifies the following risk profile for those experiencing clinically relevant difficulties lasting longer than a month (8% of the sample). Variables most associated with clinically relevant difficulties were lower emotional stability, higher anxiety, higher depression, higher severity and disruption of difficulties, and higher proportion seeking external support.

These findings are higher than some other naturalistic surveys, such as Simonsson et al.^7^ and Olofsson et al.^4^, who found 8.9% of people and 6.4% of people reported functional impairment lasting longer than a day after psychedelic use (as compared to this study, in which 48.3% reported difficulties lasting longer than a day, and 6% experienced moderate-to-severe difficulties lasting longer than a month). The high prevalence of extended difficulties in this study are closer to the estimates found by Nayak et al. (7% reported difficulties two months after psilocybin use); Weiss et al.^9^ (14% of people reported feeling more anxious for an extended period due to psychedelic usage); Kvam et al.^8^ (23% reported persistent adverse events after psychedelic use) and Davis et al.^10^ survey of veterans (59% reported adverse events). The range of prevalence outcomes in surveys of post-psychedelic difficulties could be caused by different demographics and different wording of questions.

MDMA was the only drug that was not endorsed by respondents as being specifically associated with extended difficulties. Moreover, the percentages of those having difficulties for over a month with disruptions vs not (Fig. 5) was approximately equivalent for MDMA, but for other drugs the percentage reporting difficulties for over a month with disruptions was greater than those who did not. These data might suggest a decreased odds of very long and disruptive adverse effects for MDMA compared to other psychedelics, although these data are preliminary. Future research should examine this possibility in more detail.

The specific types of difficulties reported, most notably existential struggle (37%) and depression (34%), reflect the profound psychological shifts often catalysed by psychedelic substances and reflect the persisting emotional and existential challenges primarily reported in smaller surveys ^5,6^. Interestingly, while existential struggle was the most common long-term difficulty (especially in Asia) and was rated as the most severe, it was also perceived as the most significant contributor to a healing process. This fits with the interview-based findings of Argyri et al.^24^, which found existential distress as a catalyst for meaning-making and transformative learning. It also aligns with Evans et al.^5^, where 90% of respondents felt benefits outweighed risks in supportive environments. There were minimal continental differences between symptom reporting, with the exception of relatively high rates of existential struggle with respondents in Asia, although variations in continental sample sizes may partially account for differences, and differences may not be especially representative, or they may represent genuine cultural or linguistic differences in experience or interpretation.

Those with clinically relevant difficulties in the current study were more likely to be younger at the time of their first psychedelic use experience, consistent with Bremler et al.^22^ findings, suggesting that neuroplasticity or psychological immaturity in younger users may increase vulnerability ^25^. Furthermore, the association with lower Emotional Stability (Neuroticism) supports the findings of Barrett et al.^15^. A significant finding is the link between pre-existing mental health conditions and extended difficulties, which fits with the findings of Bremler et al.^22^, Evans et al.^5^, and Olofsson et al.^4^.

Participants using psychedelics to treat depression or anxiety were more likely to report prolonged disruptive outcomes. This validates the clinical need for strong screening and safety guidelines, particularly for populations with Major Depressive Disorder (MDD) or Generalized Anxiety Disorder (GAD). Although these individuals seek psychedelics for relief, they may be at higher risk for the “causal” induction of further distress, however these data may also alternatively reflect a greater degree of ongoing distress with this demographic.

These data underscore the role of the broader psychosocial environment as a moderator of the effects of psychedelics. Those with disruptive difficulties reported lower family support and were more likely to perceive a link between their distress and the specific setting of their use. This supports findings from Robinson et al.^6^ and Simonsson et al.^7,26^ regarding the dangers of unguided environments or a lack of preparation.

Finally, 42% of participants with disruptive difficulties lasting a month or more sought external support to help deal with it, and 79% found that support to be effective, although Robinson et al.^6^ survey of coping strategies indicates that some symptom types respond better than others to such external support, and differentially to different sources. We do not know what sort of support respondents sought – therapy, psychiatry, peer support – but the ‘success rate’ suggests these difficulties respond to interventions, and such interventions need to be investigated and made more widely available^24,27,1,28,6^ .

### Limitations and Future Research

This study has several limitations. First, as a retrospective survey, it is subject to recall bias; participants may not accurately estimate the duration or severity of difficulties that occurred years prior. Second, the sample is self-selected, and those that have had particularly salient experiences (positive or negative) with psychedelics may be more motivated to participate, potentially skewing prevalence rates. The use of online recruitment via NGOs and social media may over-represent “psychedelic-literate” communities, which could explain the high perceived psychedelic knowledge scores and the general pro-legalization stance of the sample. Additionally, whereas the survey was translated into 19 languages, the back-translation process relied partly on Google Translate before expert review, which may have introduced subtle linguistic inaccuracies in nuanced psychological terms. Finally, the study is correlational, meaning we cannot state whether psychedelics caused the difficulties or if they exacerbated pre-existing conditions.

It should be noted that the findings presented here on extended difficulties are arguably among the most well-powered and globally representative to-date, given the multilingual and internationally collaborative nature of the study. Nonetheless, that only 71% of the 9,087 overall survey respondents completed the extended difficulties survey subsection reported here may have skewed the rates via selection bias. Nevertheless, it underscores that continued globally collaborative efforts are needed to elucidate the nature and prevalence of post-psychedelic difficulties.

Future studies should prioritize prospective, longitudinal designs that follow users from preparation through long-term integration. This would allow for a more accurate assessment of causality versus the potential effects of pre-existing conditions. Research should also continue to investigate why existential struggle is uniquely associated with both high severity and high healing potential, perhaps through qualitative interviews. Another important future avenue is the development and testing of treatment protocols for post-psychedelic difficulties. This study suggests external support helps 80% of those who experience disruptive difficulties lasting longer than a month, but more research is needed on the most effective forms of external support for different sorts of difficulties.

## Conclusion

Using data from a large and globally diverse survey of people who use psychedelics, this study demonstrates that extended post-psychedelic difficulties are heterogeneous and clinically meaningful for a substantial minority of users. Although many individuals experience transient challenges that are perceived as part of a longer-term healing process, approximately 6% report difficulties lasting a month or longer that significantly interfere with daily functioning. The experiences are associated with identifiable demographic and psychological, and contextual risk factors, underscoring the need for continued research on harm reduction with psychedelic substances, evidence-based education, and expanded support services to both reduce preventable risks and to treat and mitigate them when they occur.

## Supplementary Material

This is a list of supplementary files associated with this preprint. Click to download.

• GlobalPsychedelicSurvey2025EN02MAY2025.pdf

## Figures and Tables

**Table 1:** Variables and measures included in analyses

***Demographics***	Age
	Biological sex
	Relationship status
	How old when first tried psychedelics
	Level of education
	Household income
	Continent of domicile: Europe, North America, South America, Asia, Oceania, Other (‘Other’ category not included in our analysis). The groups are based on the United Nations’ Geoscheme, a regional classification system that divides 248 countries into continental regions representing Africa, the Americas, Asia, Europe, and Oceania.
***Personality and Mental health***	Ten Item Personality Inventory (7-point Likert scale from Strongly Disagree to Strongly Agree)
	Anxiety (GAD) (4-point response scale from *Not at All* to *Nearly Every Day*)
	Depression (PHQ-9) (4-point response scale from *Not at All* to *Nearly Every Day*)
***Attitudes and use of psychedelics***	Level of knowledge of psychedelics (response scale from 0 *No Knowledge at All* to 10 *Extremely Knowledgeable*)
	Level of experience of psychedelics (response scale from 0 *No Experience at All* to 10 *Extremely Experienced*)
	Partner’s / Friend’s / Family’s view of using psychedelics (5-point scale from *Strongly Opposed* to *Strongly Supportiv*e, plus *Not Applicable* option)
	Support of legal sale of psychedelics (response scale from 0 *Strongly Oppose* to 10 *Strongly Support*)
***Extended difficulties and related questions***	Use of psychedelics for mental health, physical health or problematic behaviour
	Extended difficulties experienced after a psychedelic episode, and duration of these difficulties. Types of difficulties and duration points are listed in Table 3.
	Perceived severity (5-point scale from Not At All Severe to Extremely Severe), disruption to daily life (5-point scale from Not At All to Completely), and perceived contribution to healing of difficulties (5-point scale from Not At All to Completely)
	Psychedelic drugs and settings typically associated with extended difficulties
	Having gained external support for difficulties or challenging experiences, and perceived benefits of this

**Table 2:** Descriptive statistics pertaining to the types of extended difficulties reported in the GPS (as a percentage of N = 6476)

A sense of disconnection from other people	Difficulty of any duration	Duration > 24 hours and < 1 week	Duration > 1 week and < 1 month	Duration > 1 month and < 1 year	Duration > 1 year	Severity rating (mean)	Disruption to life rating (mean)	Contribution to healing (mean)
	26.8%	17.9%	4.4%	2.6%	1.9%	1.86	1.89	2.72
Anxiety and/or panic attacks	18.6%	10.6%	3.3%	2.6%	2.1%	2.23	2.26	2.58
Feelings of depression	34.0%	**24.2%**	5.0%	2.8%	1.9%	2.05	2.12	2.62
Existential struggle	**36.6%**	19.7%	**7.4%**	**4.8%**	**4.7%**	**2.33**	2.06	**3.31**
Derealization	29.4%	18.5%	4.8%	3.2%	2.9%	1.87	1.75	2.68
Depersonalization	25.6%	16.6%	4.0%	2.6%	2.4%	1.91	1.78	2.72
Lowered self-esteem	17.7%	10.3%	2.8%	2.4%	2.2%	2.34	**2.31**	2.67
Sleep problems inc. nightmares	19.7%	13.7%	2.9%	1.7%	1.5%	2.07	2.04	1.98
Cognitive confusion or memory issues	16.9%	11.8%	2.3%	1.4%	1.4%	1.74	1.93	1.82
Visual distortions	14.4%	8.8%	1.9%	1.6%	2.1%	1.60	1.42	1.84
Disappointment with events after the experience	11.7%	6.0%	2.1%	1.7%	1.9%	2.12	1.99	2.41
Any of the difficulty types	48.3%	40.8%	31.6%	18.5%	9.9%	1.79	1.71	2.59

Note: Highest prevalence for each column highlighted in bold and grey shading for ease of visual reference

**Table 3:** Prevalence of extended difficulty types across continent geographic regions (shown as percentages of N = 6410)

Social disconnection	Europe N = 1882	N. America N = 2720	S. America N = 1121	Asia N = 501	Oceania N = 186	Chi Square	Cramer’s V	Sig
	29.1%	23.5%	32.2%	23.6%	26.9%	39.8	0.08	0.001
Anxiety and panic attacks	18.8%	18.4%	16.9%	21.8%	18.8%	5.68	0.03	0.225
Feelings of depression	37.2%	31.3%	36.3%	34.9%	25.8%	25.7	0.06	0.001
Existential struggle	33.0%	35.1%	38.9%	53.9%	32.8%	81.2	0.11	0.001
Derealisation	30.4%	26.9%	31.0%	36.9%	25.3%	25.7	0.06	0.001
Depersonalisation	24.9%	23.8%	28.5%	31.7%	23.7%	20.4	0.06	0.001
Lowered self esteem	19.0%	14.9%	21.3%	19.0%	19.9%	28.1	0.07	0.001
Sleep problems	20.5%	18.4%	21.7%	21.8%	13.4%	12.5	0.04	0.01
Cognitive confusion or memory problems	17.7%	14.1%	19.4%	21.8%	18.3%	30.7	0.07	0.001
Visual distortions	13.8%	14.6%	12.0%	19.6%	16.1%	17.5	0.05	0.002
Disappointment with events	11.5%	11.7%	8.8%	20.0%	8.6%	43.8	0.08	0.001

**Table 4:** T-tests comparing “disruptive difficulties lasting over a month” vs. “other difficulties” groups on selected continuous variables

Variable	N	Mean for those with difficulties >month +disruption (N = 547)	Mean for other participants (N = 5929)	Cohen’s *d*	*t* (t-test)	*df*	*p* value
Age	6476	37.52	43.12	.404	9.83	675	.001
How old when first tried psychedelics	6476	22.90	26.43	.299	8.28	726	.001
Level of education	6476	3.92	4.14	.174	3.89	643	.001
Household income	6476	2.80	3.06	.289	6.46	646	.001
Level of knowledge of psychedelics	6476	7.15	7.37	.113	2.38	636	.018
Level of experience of psychedelics	6476	6.41	6.35	−.026	−.59	655	.551
Partner’s view of using psychedelics (higher score = more supportive)	6467	5.31	5.26	−.027	−.54	627	.588
Family’s view of using psychedelics (higher score = more supportive)	6469	3.81	4.31	.236	4.90	633	.001
Friends’ view of using psychedelics (higher score = more supportive)	6469	4.22	4.42	.169	3.22	616	.001
Big Five: Conscientiousness	6463	4.83	5.29	.330	7.11	642	.001
Big Five: Agreeableness	6463	4.91	5.28	.317	7.03	649	.001
Big Five: Emotional Stability	6463	3.90	4.74	.573	12.72	649	.001
Big Five: Openness to Experience	6463	5.84	5.89	.050	1.07	638	.284
Big Five: Extraversion	6463	3.86	4.02	.105	2.30	649	.020
Anxiety level (GAD)	6460	8.20	4.59	−.753	−13.6	607	.001
Depression level (PHQ-9)	6460	8.88	4.72	−.865	−.15.2	603	.001
Mean severity of difficulties reported	4396	2.94	1.63	−1.816	−37.7	688	.001
Mean disruption to life of difficulties reported	4396	3.03	1.53	−2.368	−45.8	662	.001
Mean contribution to healing of difficulties	4396	2.94	2.54	−.329	−7.5	729	.001
Support of legal sale of psychedelics to adults (1 to 10, high score = higher support)	7473	8.33	7.68	.256	4.89	606	.001

**Table 5:** Associations between “disruptive difficulties lasting over a month” and selected categorical variables

Variable	N	Difficulties >month +disruption (%) (N = 547)	Other participants (%) N = 5929)	*Chi Square*	*Cramer’s V*	*df*	*^p^* value
% Biological female	6476	53.4	49.4	0.75	.02	1	.080
% Single, never married	6476	41.5	30.6	41.5	.08	5	.001
% Who have sought external support for difficulties	5991	42.4	27.0	55.5	.09	1	.001
% Who did get support and found that external support helpful	1696	79.1	93.6	52.6	.18	1	.001
% Use psychedelics for treating a mental health condition	6476	58.7	41.4	60.8	.10	1	.001
% Use psychedelics to treat a physical health condition	6476	15.5	10.1	16.0	.05	1	.001
% Use psychedelics to reduce use of other substance	6476	18.3	11.2	24.4	.06	1	.001
% Use psychedelics for problematic behaviour	6476	26.3	13.6	64.8	.10	1	.001
% Report encountering an “entity” i.e. an apparently autonomous non-physical being, while on psychedelics	5634	52.6	43.6	17.4	.06	1	.001
Was a speciffic psychedelic associated with ongoing difficulties? (11x2 Chi Square)	4370	See Fig. 1		111.9	.16	14	.001
Was a specific setting associated with difficulties? (7x2 Chi Square)	4370	See Fig. 1		105.7	.16	6	.001

## Data Availability

Data is available upon request.
